# Responses of FEV_6_, FVC, and FET to inhaled bronchodilator in the adult general population

**DOI:** 10.1186/1465-9921-10-71

**Published:** 2009-07-28

**Authors:** Annette Kainu, Ari Lindqvist, Seppo Sarna, Bo Lundbäck, Anssi Sovijärvi

**Affiliations:** 1 Division of Pulmonary Medicine, Department of Medicine, Helsinki University Central Hospital, PO Box 340, FIN-00029 HUS, Helsinki, Finland; 2 Research Unit of Pulmonary Diseases, Department of Medicine, Helsinki University Central Hospital; 3 Clinical Research Institute Ltd, Helsinki, Finland; 4 Department of Public Health, University of Helsinki, Helsinki, Finland; 5 Department of Internal Medicine/Respiratory Medicine and Allergology, Sahlgrenska Academy, University of Gothenburg, Sweden; 6 Division of Clinical Physiology and Nuclear Medicine, Laboratory Department, Helsinki University Central Hospital, Helsinki, Finland

## Abstract

**Background:**

The assessment of bronchodilator-induced change in forced vital capacity (FVC) is dependent on forced expiratory time (FET) in subjects with airflow limitation. Limited information is available on the concurrent responses of FVC, forced expiratory volume in six seconds (FEV_6_), and FET in the bronchodilation test among patients with obstructive airways disease or in the general population. The aim of this study was to assess the changes in FEV_6_, FVC, and FET, and their relationships in a standardized bronchodilation test in the general population.

**Methods:**

We studied bronchodilation response in a general adult population sample of 628 individuals (260 men, 368 women) with flow-volume spirometry. The largest FVC, the corresponding FET and the largest FEV_6 _both at the baseline and after 0.4 mg of inhaled salbutamol were selected for analysis.

**Results:**

After administration of salbutamol FEV_6 _decreased on average -13.4 (95% CI -22.3 to -4.5) ml or -0.2% (-0.4% to 0.0%) from the baseline. The 95^th ^percentile of change in FEV_6 _was 169.1 ml and 5.0%. FVC decreased on average -42.8 (-52.4 to -33.3) ml or -1.0% (-1.2% to -0.7%). Concurrently FET changed on average -0.2 (-0.4 to 0.0) seconds or 0.4% (-1.4% to 2.3%). There were four subjects with an increase of FVC over 12% and only one of these was associated with prolonged FET after salbutamol. Changes in FEV_6 _and FVC were more frequently positive in subjects with reduced FEV_1_/FVC in baseline spirometry.

**Conclusion:**

In general adult population, both FEV_6 _and FVC tended to decrease, but FET remained almost unchanged, in the bronchodilation test. However, those subjects with signs of airflow limitation at the baseline showed frequently some increase of FEV_6 _and FVC in the bronchodilation test without change in FET. We suggest that FEV_6 _could be used in assessment of bronchodilation response in lieu of FVC removing the need for regulation of FET during bronchodilation testing.

## Introduction

In recent years forced expiratory volume in six seconds (FEV_6_) has evolved as a novel parameter in flow-volume spirometry that has been suggested to replace forced vital capacity (FVC) for some clinical applications [1-4]. A practical benefit of using FEV_6 _would be easier performance by patients because maximal end-expiration can be avoided. This measure could especially lend itself for use in the primary care setting [1]. Reference values and data on reliability and utility in the diagnosis of obstructive and restrictive lung diseases are emerging for FEV_6 _[2-9].

Bronchodilation induced by pharmacological agents is an important feature of asthma, whereas chronic obstructive pulmonary disease (COPD) is characterized by chronic airflow limitation that is not fully reversible [10]. In COPD, bronchodilation response may be reflected as increase of FVC, as an indicator of relief of air trapping [11,12]. The FVC manoeuvre is technically demanding, significantly affected by expiratory time in subjects with airflow limitation, and sensitive to the impact of tiring [13].

The joint American Thoracic Society (ATS) and European Respiratory Society (ERS) Task Force on Standardisation of Lung Function Testing recommended in 2005 that a 12% and 200 ml improvement in either FEV_1 _or FVC from baseline would be considered a significant bronchodilation response [14]. Recently we have shown that FEV_1 _response to bronchodilation by around 9% from the baseline in an adult urban population is significant [15]. If bronchodilation response is observed only in FVC, the concurrent change in forced expiratory time (FET) should be evaluated [14]. Based on the observed intra-session variability of FET in the general population, we have suggested that 3 seconds would constitute a significant change [16]. In subjects with airflow limitation the intra-session repeatability of FEV_6 _was at least equal to the repeatability of FVC [16]. FEV_6 _is the least variable of the FEVx [17]. Both FEV_3 _and FEV_6 _have been shown to increase significantly when the increase in FVC was not caused by longer exhalation time [18]. Standardisation of FET during bronchodilation testing is problematic. Since FEV_6 _would offer better repeatability and an unequivocal end-of-test measure, it would be interesting to assess FEV_6 _as a surrogate measure of FVC response in the bronchodilation test.

The aim of this study was to evaluate the concurrent changes in FEV_6_, FVC, and FET in a standardized bronchodilation test and their association with airflow limitation in a general adult population sample using flow-volume spirometry. Furthermore, the role of FEV_6 _as a surrogate of FVC in the bronchodilation test was evaluated.

## Materials and methods

### Subjects

In 1995 the original sample of 8000 adults aged 20–69 years was randomly selected from the Finnish Population Registre Center to represent the adult population of Helsinki, Finland. Randomization was stratified by gender and 10-year age cohorts. In phase I a postal questionnaire study was completed, with 6062 responders. In 2000 a further random sample of 1200 subjects were sampled from the original postal questionnaire responders to a subsequent clinical study. During 2000–2003 a total of 643 subjects participated in phase II of the FinEsS-Helsinki study. The study protocol has been reported in previous articles [15,16,19].

In this study, 628 acceptable spirometric measurements (260 for men, 368 for women) with bronchodilation testing and a structured interview were completed. Study subjects were interviewed using a structured questionnaire to obtain information about general health, respiratory illnesses, medications and environmental exposures. The Finnish FinEsS structured interview has been developed from the OLIN questionnaire [20,21].

The study was conducted according to the Helsinki Declaration and approved by the Ethics Committee of the Department of Medicine of Helsinki University Central Hospital. All participants gave informed consent.

### Measurements

Spirometry procedures were based on the 1994 recommendation of the American Thoracic Society [22], with the exception of repeatability criteria that were based on modified ERS criteria [23]. The two largest FEV_1 _and FVC were required to be within 150 ml or 5% of the respective volume, whichever was greater, and the two largest peak expiratory flows (PEF) were required to be within 10%.

Spirometry was completed with a flow-volume spirometry device (VMax 20c, Sensor Medics, Yorba Linda, CA, USA) with the patient seated. Each subject performed three to eight forced expiratory manoeuvres and inspiratory spirograms were recorded in conjunction with expiratory spirograms whenever feasible. In the bronchodilation test the subjects inhaled 0.4 mg of salbutamol aerosol (Ventoline 0.2 mg, GlaxoSmithKline, London, UK) through a spacer device (Volumatic, GlaxoSmithKline, London, UK) on two separate doses and spirometry was repeated after 15 minutes rest. Spirometry was evaluated using the current Finnish reference values, which do not yet contain reference values for FEV_6 _[24]. The detailed spirometry procedure has been published previously [15,16].

The spirometry variables evaluated in this study were FVC, FEV_6 _and FET. The largest FEV_6 _and FVC from the acceptable pre- and post-bronchodilation curves, and the FET measured from the curve with the largest FVC were selected for analysis. The FET defined and recorded by the spirometer software was used (Vision Software 05-2A, Vmax System, Sensor Medics, Yorba Linda, CA, USA). The beginning point of measurement is the back extrapolated time zero [14,25] and the end-point at the beginning of the end-expiratory plateau. In cases where the total exhalation time recorded by the spirometer was under six seconds, the FVC was used in lieu of FEV_6_. The difference between baseline spirometry and post-bronchodilator spirometry was assessed using both absolute change and change relative to baseline spirometry.

The smoking history was evaluated from the structured interview and subjects were categorized into never-smokers, former smokers and current smokers in addition to calculating smoking pack-years for the ever-smokers. Former smokers were required to have stopped smoking at least 12 months prior to the study. Previously published criteria based on the structured interview were used to define a subgroup of healthy asymptomatic non-smokers [15,20] to assess the upper limit of normal. The anthropometric parameters analysed were gender, age, height, weight, and body mass index (BMI, kg/m^2^). The descriptive statistics of the study population including baseline spirometric results and smoking history are outlined in Table 1.

**Table 1:** Anthropometric and baseline spirometric statistics of the study population

	men (n = 260)			women (n = 368)		
	mean (SD)	95% CI	range	mean (SD)	95% CI	range
age [yrs]	48.6 (12.7)	47.0–50.1	26.3–74.2	49.5 (13.2)	48.2–50.9	25.7–74.4
height [m]	1.78 (0.07)	1.77–1.79	1.62–1.98	1.64 (0.06)	1.63–1.64	1.46–1.83
weight [kg]	83.9 (14.1)	82.2–85.6	52.4–139.0	68.8 (13.7)	67.4–70.2	44.0–133.0
BMI [kg/m^2^]	26.5 (4.3)	26.0–27.0	17.1–44.9	25.7 (5.1)	25.2–26.2	16.9–53.3
baseline FVC [l]	5.075 (0.911)	4.964–5.187	2.182–8.033	3.549 (0.660)	3.482–3.617	2.013–5.388
baseline FVC % of reference*	98.1 (12.3)	96.6–99.6	50.8–131.2	99.5 (12.5)	98.2–100.7	71.9–144.6
baseline FEV_1 _[l]	3.897 (0.832)	3.795–3.999	1.016–5.895	2.784 (0.591)	2.724–2.845	0.992–4.495
baseline FEV_1 _% of reference*	92.9 (14.9)	91.0–94.7	29.3–128.7	94.7 (13.1)	93.3–96.0	40.6–132.8
baseline FEV_6 _[l]**	4.888 (0.937)	4.774–5.002	2.14–7.75	3.437 (0.675)	3.368–3.506	1.84–5.31
baseline FET [s] ±	11.5 (4.3)	11.0–12.1	1.6–37.0	10.5 (4.5)	10.0–10.9	1.7–36.8
baseline FEV_1_/FVC [%]	76.5 (7.8)	75.5–77.5	34.8–96.3	78.3 (7.0)	77.6–79.0	44.7–92.9
baseline FEV_1_/FVC % of reference*	94.5 (9.6)	93.4–95.7	42.6–121.2	95.3 (8.0)	94.5–96.1	55.1–114.9
		pack-years mean (SD)	range		pack-years mean (SD)	range
non-smokers†	n = 112	n.a.	n.a.	n = 226	n.a.	n.a.
former smokers	n = 59	23.6 (16.9)	5.5–82.5	n = 49	18.0 (13.7)	1.2–66.0
current smokers	n = 89	26.2 (22.0)	0.4–95.0	n = 93	19.1 (16.0)	0.6–86.0

BMI = body mass index; FVC = forced vital capacity; FEV_1 _= forced expiratory volume in one second; FEV_6 _= forced expiratory volume in six seconds; FET = forced expiratory time; n.a. = not applicable

* predicted values from [24]

** maximum value of acceptable curves

± value corresponding to largest FVC measurement

† including former smokers smoking under 5 pack-years and smoking cessation over 5 years previously

### Analysis

Statistical analyses were performed with SPSS for Windows (version 15.01; SPSS, Chicago, IL, USA). Distribution of parameters was assessed using scatter graphs and normality was assessed with the Kolmogorov-Smirnov test for normality. Pearson correlation coefficient (r) was used to assess the correlation of changes in FVC and FEV_6 _with FET. The intra-class correlation coefficient was used to assess the agreement between change in FVC and FEV_6 _using a one-way random effects model [26,27]. The intra-class correlation coefficient produces measures of consistency or agreement of values within cases. Analysis of variance (ANOVA) was used for categorical comparisons. The method described by Bland & Altman [28] was modified to demonstrate dependence of change in FVC and FET of their respective average values [29]. P-values less than 0.05 were considered statistically significant for all other analyses, except for correlations, for which a p-value less than 0.01 was regarded significant. All analyses were two-sided unless otherwise indicated.

## Results

**Figure 1:**
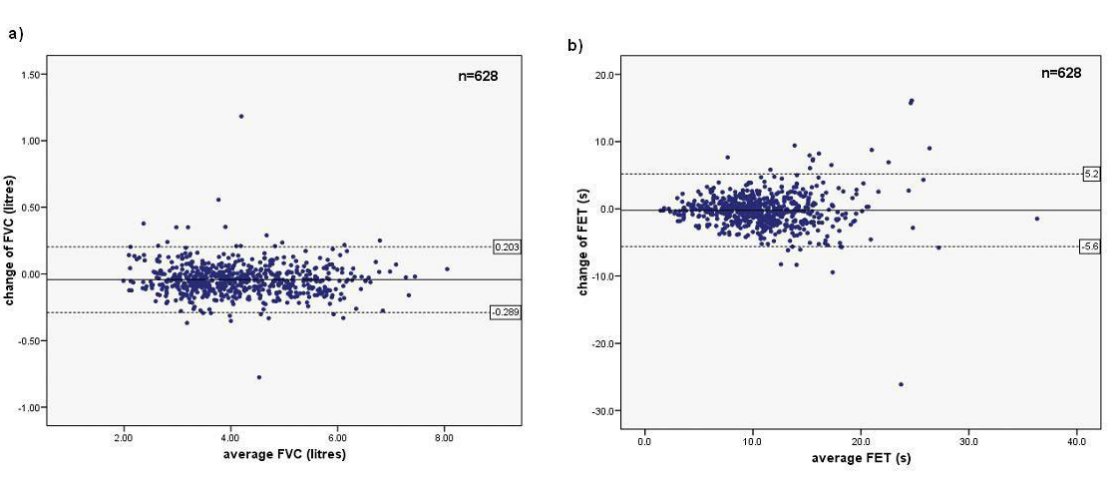
**Bland & Altman graphs depicting: a) individual changes in forced vital capacity (FVC) in relation to average FVC, and b) individual changes in forced expiratory time (FET) in relation to average FET, in a bronchodilation test with salbutamol aerosol 0.4 mg in the general adult population (n = 628)**. The dotted lines indicate the 2 SD limits from the respective mean value. Larger bronchodilation responses in FVC were seen in subjects with lower FVC and larger variation in FET with prolonged FET, which was associated to more severe obstruction.

Changes in FVC and FEV_6 _in bronchodilation test were normally distributed. The concurrent changes in FEV_6_, FVC and FET in the bronchodilation test are outlined in Table 2. FEV_6 _decreased statistically significantly more in women both in absolute and in relative terms, whereas the gender difference was only significant in relative change in FVC. Change of FVC and FET in relation to their respective average values are shown in Figure 1 in a modified Bland-Altman plot. At higher FVC there were less bronchodilation changes, but at higher FET more frequent changes associated with both underlying airflow limitation and poorer repeatability of FET in comparison to FVC. In the bronchodilation test 23.1% of men and 33.2% of women had a decrease of FVC greater than 2.5% from the baseline. The mean change in FVC was -42.8 (95% CI -52.4 to -33.3) ml or -1.0% (-1.2% to -0.7%). The upper 95^th ^percentile for change in FVC was 137.0 ml and 4.0%. The mean change in FET was -0.2 (-0.4–0.0) seconds or 0.4% (-1.4%–2.3%), with a 95^th ^percentile of 3.4 seconds or 44.0%.

**Table 2:** Concurrent changes in forced expiratory volume in one (FEV_1_) and six (FEV_6_) seconds, forced vital capacity (FVC) and forced expiratory time (FET) in the bronchodilation test in general population

	men (n = 260)			women (n = 368)			
	mean (SD)	95% CI	95th percentile	mean (SD)	95% CI	95th percentile	gender difference (p value)
change of FEV_1 _[ml]	107.4 (130.6)	91.4 – 123.3	335.3	55.9 (86.2)	47.1 – 64.8	214.5	<0.001
change of FEV_1 _% from baseline	3.0 (4.3)	2.5 – 3.5	8.6	2.2 (3.7)	1.8 – 2.6	8.4	0.009
change of FEV_6 _[ml]	-2.1 (137.7)	-18.9 – 14.8	195.8	-21.4 (93.5)	-31.0 – -11.8	144.9	0.036
change of FEV_6 _% from baseline	0.1 (3.5)	-0.3 – 0.6	4.8	-0.5 (3.1)	-0.8 – -0.2	5.1	0.023
change of FVC [ml]	-35.6 (147.6)	-53.6 – -17.6	170.2	-48.1 (100.9)	-58.4 – -37.8	132.2	n.s.
change of FVC % from baseline	-0.6 (3.4)	-1.0 – -0.2	3.6	-1.2 (3.2)	-1.6 – -0.9	4.4	0.016
change of FET [s]	-0.1 (2.6)	-0.5 – 0.2	3.2	-0.3 (2.7)	-0.5 – 0.0	3.7	n.s.
change of FET % from baseline	-0.6 (21.2)	-3.2 – 2.0	34.7	1.2 (25.6)	-1.4 – 3.8	48.6	n.s.

FEV_t _= forced expiratory volume in t second(s), FVC = forced vital capacity; FET = forced expiratory time; CI = confidence interval; n.s. = not significant

**Figure 2:**
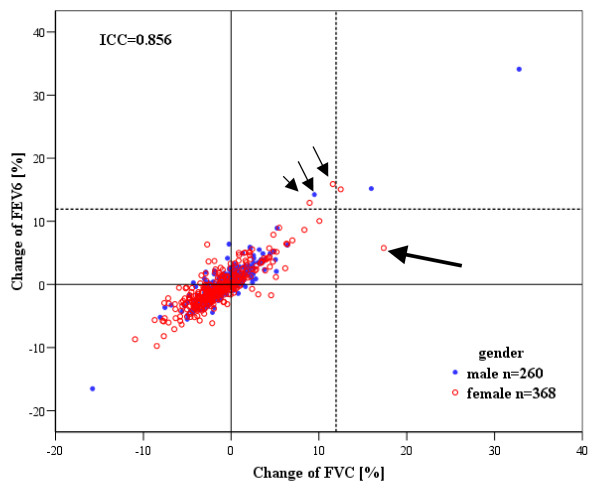
**Individual changes of forced vital capacity (FVC) and forced expiratory volume in six seconds (FEV_6_) after bronchodilation in the general adult population (n = 628)**. The dotted lines represent the significant change limit of 12% from the baseline [14] for FVC. All subjects with a change over 12% also fulfilled the absolute change criterion of 200 ml. The thick arrow highlights one subject with prolonged post-bronchodilation FET resulting in increase of FVC in the absence of significant change in FEV_6_. The small arrows show subjects with significantly improved FEV_6 _in the absence of increase in FVC, caused by shorter exhalations in post-bronchodilation spirometry. ICC = intra-class correlation.

FEV_6 _tended to decrease during bronchodilation test, but the mean reduction was less marked than in FVC (mean change -13.4 (-22.3 to -4.5) ml or -0.2% (-0.4%–0.0%)). The 95^th ^percentile for change in FEV_6 _was 169.1 ml and 5.0%. The individual concurrent changes in FEV_6 _and FVC are demonstrated in Figure 2. The intra-class correlation coefficient (ICC) for the concurrent absolute change in FEV_6 _and FVC was 0.84 (0.82–0.87) and for the relative change 0.86 (0.83–0.88). The agreement between changes in FEV_6 _and FVC was better in subjects with airways obstruction (FEV_1_/FVC post-bronchodilator < LLN); ICC for the relative change in subjects with obstruction was 0.91 (0.84–0.95) and in those who were non-obstructed 0.80 (0.77–0.83). Age, height, weight or BMI did not correlate significantly with changes in FVC, FEV_6 _or FET during the bronchodilation test.

There were four subjects with the increase of FVC from the baseline at least 12% and 200 ml, yielding a population prevalence of 0.6% for a significant improvement of FVC in the bronchodilation test. Using the same threshold values six subjects showed a significant change of FEV_6_. The changes in FVC, FEV_6_, FEV_1 _and FET of these subjects are individually shown in Table 3. One subject had a significant increase in FVC, but an insignificant increase in FEV_6_; for her the increase of FVC was associated with an increase of FET by 8 seconds and 71% relative to the baseline FET. Three subjects had a significant increase in FEV_6 _but a smaller increase in FVC, which were associated to shorter FET in post-bronchodilator spirometry.

**Table 3:** Data of subjects with significant changes in forced expiratory volume in six seconds (FEV_6_) or forced vital capacity (FVC) in the bronchodilation test

age	gender	FEV_1_/FVC post	FEV_1_ post	smoking status	history of OAD	change in FVC		change in FEV_6_		change in FET		change in FEV_1_	
[yrs]		[%]	[%*]	[pack-years]		ml	%	ml	%	s	%	ml	%
52.9	m	48.9%	62.1%	current smoker 22.2	asthma	**1183**	**32.8**	**979**	**34,1**	0.3	1.5	**573**	**32.4**
50.5	w	55.5%	59.7%	current smoker 33.0	no dg no med	350	11.6	**424**	**15.9**	-1.2	-10.1	**381**	**25.5**
67.8	m	44.7%	51.4%	former smoker 35.0	COPD	**557**	**16.0**	**446**	**15.2**	2.9	22.1	**321**	**21.6**
47.5	w	50.0%	51.9%	current smoker 52.2	asthma	**350**	**12.5**	**336**	**15.1**	-1.7	-9.2	**246**	**18.5**
63.0	m	38.7%	46.9%	current smoker 84.0	no dg asthma med	353	9.5	**418**	**14.2**	-1.3	-7.8	**241**	**18.1**
52.6	w	78.4	79.3	non-smoker 0.0	asthma	240	8.9	**329**	**12.9**	-4.5	-34.5	**364**	**18.8**
52.4	w	43.1%	45.0%	former smoker 14.0	asthma	**378**	**17.4**	107	5.8	**8.0**	**70.7**	109	11.0

FEV1 = forced expiratory volume in one second; FVC = forced vital capacity; OAD = obstructive airways disease; FEV6 = forced expiratory volume in six seconds; FET = forced expiratory time; dg = diagnosis; med = medication.

* of predicted values from [24]

**Figure 3:**
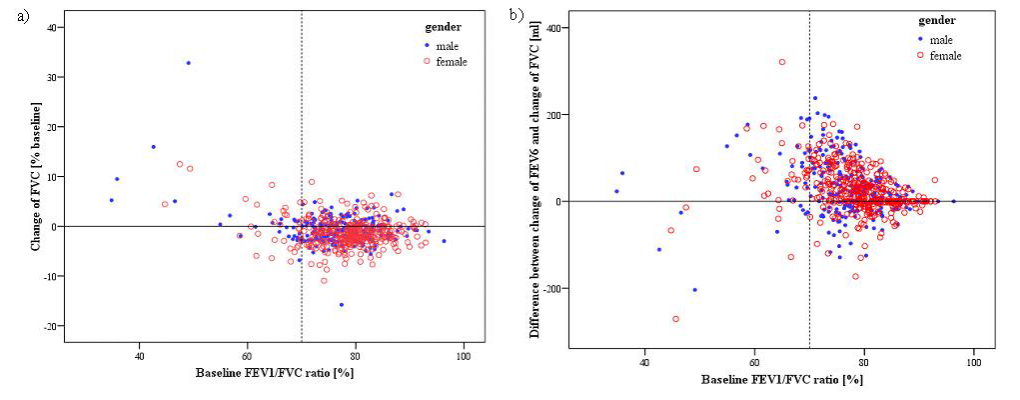
**The relationship of a) individual changes in forced vital capacity (FVC) after bronchodilation, and b) the difference between the individual changes in forced expiratory volume in six seconds (FEV_6_) and FVC, to the ratio of forced expiratory volume in one second (FEV_1_) to FVC at the baseline in general adult population (n = 628)**. The dotted line indicates the FEV_1_/FVC limit for airflow obstruction (0.7). In subjects with airflow obstruction, positive FVC bronchodilation response was more common and the difference between FVC and FEV_6 _responses tended to be larger.

The relationship of individual changes of FVC and the difference between changes in FEV_6 _and FVC in bronchodilation test, to baseline FEV_1_/FVC is demonstrated in Figure 3. Increase of FVC in the bronchodilation test associated inversely with FEV_1_/FVC ratio and FEV_1_. The changes in flow-volume spirometry variables stratified by FEV_1_/FVC ratio at the baseline below or above lower limit of normal (LLN) are shown in Table 4. In subjects with airflow limitation the changes in FEV_6 _and FVC were on the average +2.4% and +0.8%, respectively. In subjects with no airflow limitation the corresponding values were -0.7% and -1.3%. Conversely, there was no significant difference in FET between the subjects with or without airflow limitation at the baseline.

**Table 4:** Change in flow-volume spirometry variables in subjects with and without airflow limitation at the baseline in the population sample

	FEV_1_/FVC ≥ LLN* (n = 537) mean (SD)	FEV_1_/FVC < LLN* (n = 91) mean (SD)	p value
change of FEV_1 _(ml)	66.2 (99.9)	142.6 (139.1)	p < 0.001
(% from baseline)	1.9 (2.9)	6.2 (6.4)	p < 0.001
change of FEV_6 _(ml)	-27.3 (98.0)	68.8 (160.1)	p < 0.001
(% from baseline)	-0.7 (2.5)	2.4 (5.4)	p < 0.001
change of FVC (ml)	-52.8 (103.2)	15.4 (192.3)	p < 0.001
(% from baseline)	-1.3 (2.6)	0.8 (5.6)	p < 0.001
change of FET (s)	-0.2 (2.5)	-0.4 (3.5)	p = 0.563
(% from baseline)	0.8 (24.1)	-1.6 (22.3)	p = 0.370

FET = forced expiratory time; FEV_1_ = forced expiratory volume in one second; FEV_6_ = forced expiratory volume in six seconds; FVC = forced vital capacity; LLN = lower limit of normal

* predicted values from [24]

For healthy asymptomatic non-smokers (n = 219), the 95^th ^percentile of change in FEV_6 _was 112.0 ml and 3.4%, change in FVC 92.0 ml and 2.5%, and change in FET 3.7 seconds and 50.0%.

## Discussion

The present study indicates that increase of FVC in response to inhaled salbutamol in the bronchodilation test in a general population sample is infrequent and is only rarely associated to increased expiratory time. Amongst subjects with baseline airflow limitation, FEV_6 _response differentiated those individuals by whom increase of FVC was caused by longer exhalation time. This is important, because it implies that the use of FEV_6 _would help to remove the need for standardization of exhalation time during standard bronchodilation testing. Decrease of FVC during the bronchodilation test was more frequent than previously reported, especially in healthy subjects. Therefore, the limit of significant increase of FVC might be lower than previously thought. However, the limits of any significant change are dependent of the inherent variability of the measures, like FVC, FEV_6 _and FET.

Bronchodilation response in flow-volume spirometry is primarily assessed with FEV_1_, but a significant response can also be seen in other measures, such as FVC [11-13,18,30-32]. It has been hypothesized that in patients with chronic airflow limitation combined with hyperinflation the potential increase of expiratory airflow due to bronchodilation may be attenuated in small airways due to airway-to-parenchymal interdependence [12]. The degree of obstruction in COPD has been found to modify the spirometric response to bronchodilation; FEV_1 _response shown to dominate in mild obstruction (GOLD Stage I-II) and FVC response in severe obstruction (GOLD III-IV) [11]. Also in our material from general population FVC response to bronchodilator increased as the post-bronchodilator FEV_1_/FVC ratio decreased as shown in Figure 3. However, especially in subjects with airflow limitation FVC has been found to depend on forced expiratory time; longer exhalations potentially created higher FVC values not necessarily due to actual bronchodilation [13]. At the present, FET is not routinely given in the reports of flow-volume spirometry and there is limited information on the concurrent changes of FET and FVC. It has been suggested, but not proved, that an increase in FET during bronchodilation testing in severely obstructed individuals could reflect bronchodilation in small airways [31,33].

Since a positive response in FVC is most often seen in more severe COPD in a bronchodilation test, a large part of the bronchodilation studies assessing changes in lung volumes have been conducted in groups of patients with obstructive pulmonary diseases [12,14,18,30-32]. Bronchodilating medication, its dose and mode of delivery have varied making comparisons difficult. Most studies with large number of individuals have been conducted based on patient databases, where the exclusion of untreated asthmatics is nearly impossible [31].

Differing views have been voiced on the interpretation of FVC response. In patients with marked hyperinflation, bronchodilation response has been demonstrated to occur in inspiratory capacity (IC) and residual volume (RV) with better correlation to symptom relief than with other spirometric variables [31,32]. Changes in vital capacity (VC) might in some individuals better correlate with symptomatic relief from bronchodilating medication and FVC can underestimate this volume response [34]. However, when assessing changes measured with flow-volume spirometry, FEV_6 _would offer a measure that is not influenced by as many pathophysiological factors as FVC. Increase in FEV_6 _may partially reflect flow changes also in small airways. Variables regarded to represent small airways (MEF50, MMEF) have greater inter- and intra-session variability than FEV_6 _[35,36] and are affected by concurrent changes in FVC. It is theoretically possible that in subjects with marked peripheral airways obstruction prolongation of FET after bronchodilation could imply decreased hyperinflation.

The frequent negative changes in FVC during bronchodilation test have to date been largely neglected. However, it greatly affects the distribution of values and the assessment of upper limit of normal change. We found 23% of men and 33% of women to have greater than 2.5% reduction in FVC in the bronchodilation test. One reason for reduction of FVC after the bronchodilator in healthy subjects could be the increased collapsibility of the airways as a result of the reduced airway smooth muscle tone with β2-agonists [37]. On the contrary, those subjects with markedly reduced FEV_1_/FVC ratio indicating bronchial obstruction showed increased FVC after bronchodilation in our population study (Figure 3, Table 4). In this population, the upper 95^th ^percentiles for change in FEV_6 _and FVC were around 5% and 4% from baseline, respectively. The corresponding values for the subgroup of healthy asymptomatic non-smokers were 3.4% and 2.5%.

When considering significant changes in bronchodilation testing, it is necessary to evaluate also the inherent measurement variability. In a large patient sample, the intra-session repeatability of FVC has been shown to have a 95^th ^percentile of 7.0% or 180 ml [38] although that study didn't control concurrent variations in FET either. In our earlier report from the present population sample the 95^th ^percentiles of the intra-session repeatability of FEV_6 _and FVC were 3.3% or 117 ml and 3.2% or 119 ml, respectively [16]. Concurrently FET varied on average -0.0 (2.0) seconds with a 95^th ^percentile of 2.7 seconds. Earlier, Pennock and coworkers have concluded that the within-day repeatability of FVC is 6.7% and FEV_1 _8.1% in obstructive subjects [36]. Although our results imply that an increase in FVC due to bronchodilation is statistically significant at a lower level than given in the current standards [14], the limit for significant change cannot be lower than the repeatability of the measurement.

In our study, very few positive FEV_6 _or FVC bronchodilation responses were detected in the unselected population sample, which limits the possibilities of further analysis in this study. This can partially be caused by the fact that regular medication was not discontinued for the study and hence subjects with asthma were on appropriate treatment. Since the number of subjects with positive responses was this limited in the population, concurrent changes in FEV_6_, FVC, and FET should be further evaluated in subjects with varying degrees of obstruction. However, it is clear from this unselected population study that FVC tends to decrease in the healthy subjects and thus positive changes – most likely in those with airflow obstruction – are significant at a lower level than previously thought. No differences were detected between different age-groups, but the negative changes were slightly more common in women. However, men had more airflow limitation, which was associated with more frequent positive changes in FEV_6 _and FVC during the bronchodilation test.

FEV_6 _performed in this study comparably with FVC, suggesting that it could be considered a surrogate for FVC in the bronchodilation test. FVC might underestimate changes in vital capacity (VC) in subjects with severe airflow limitation and air trapping, which has been suggested to partially account for their subjective benefit of bronchodilating medication in clinical practice [34]. In subjects with airflow limitation, change in FEV_6 _was significant in three subjects that had shorter exhalations in post-bronchodilation spirometry, which resulted in changes in FVC remaining below significant change limits (Table 3). We chose to replace FEV_6 _with FVC when FET was under 6 seconds, because the exclusion of these subjects would have created a selection bias. In subjects with FET < 6 seconds, most often healthy young adults, FVC is not dependent on FET [39]. The intra-class correlation coefficient showed good agreement between changes in FEV_6 _and FVC especially in subjects with airflow limitation in spirometry. Earlier Girard & Light have also shown that timed expiratory flows (FEV_3 _and FEV_6_) generally increase if the increase in FVC is not being caused by longer FET [13]. Previously it has been reported that FEV_6 _has the least within-session variability of the FEVx values [17] when exhalation times are over 10 seconds. It is suggested that the use of FEV_6 _would preclude the need to standardize FET and could act as a measure of bronchodilation especially in the primary care. The current standard states that FET should be analysed from those curves where the sum of FVC and FEV_1 _is the greatest [25]. Since FET varies within test session more than FVC and FEV_1 _[16], we suggest that in the bronchodilation test FET should be analysed from those curves with the largest FVC values. FEV_6 _is more repeatable than FVC also in subjects with reduced FEV_1_/FVC [15]. Earlier it has been disputed that the use of FEV_6 _could more easily misclassify subjects with borderline obstruction [4,5,7,8,40], but since positive changes in FVC in the bronchodilation test are more likely to occur in more severe airflow limitation, this should not become a problem for the use of FEV_6 _as a measure of bronchodilation in lieu of FVC.

In conclusion, we found that in a general population sample, positive FVC and FEV_6 _response to bronchodilation in flow-volume spirometry was infrequent occurring almost solely in subjects with bronchial airflow limitation. In subjects with normal FEV_1_/FVC ratio both FVC and FEV_6_, on the average, decreased. We suggest that FEV_6 _could be used in assessment of bronchodilation response in flow-volume spirometry instead of FVC; significant increase of FEV_6 _would seem to be around 6%. By using FEV_6 _for assessment of bronchodilator effect instead of FVC would remove the need for regulation of forced exhalation time during a bronchodilation test.

## Abbreviations

ATS: American Thoracic Society; ANOVA: analysis of variance; BMI: body mass index; CI: confidence interval; COPD: chronic obstructive pulmonary disease; ERS: European Respiratory Society; FET: forced expiratory time; FEVx: forced expiratory volume in × seconds; FVC: forced vital capacity; GOLD: Global Initiative for Chronic Obstructive Lung Disease; IC: Inspiratory capacity; ICC: intra-class correlation coefficient; LLN: lower limit of normal; MEF50: maximal instantaneous forced expiratory flow where 50% of FVC remains to be expired; MMEF: maximum mid-expiratory flow; OLIN: Obstructive Lung Disease in Northern Sweden Study; PEF: peak expiratory flow; RV: residual volume.

## Competing interests

The authors declare that they have no competing interests.

## Authors' contributions

AK has conducted the data processing and statistical analyses with consultative help from SS. AK has mainly drafted the text and illustrations of the article, with editorial advice from BL, AL and AS, who also have contributed to the text. All authors have read and approved the final manuscript.
